# A Reviewer Reviewed

**Published:** 1889-05

**Authors:** 


BUFFALO MEDICAL AND SURGICAL JOURNAL
A MONTHLY REVIEW OF MEDICINE AND SURGERY.

EDITORS:
THOS. LOTHROP, M. D., - - - W. W. POTTER, M. D.

All communications, whether of a literary or business character, should be addressed to the editors: 284 FRANKLIN STREET, BUFFALO, N. Y.

### Editorial.

### *A REVIEWER REVIEWED.*

### "J. C. R." AND TAIT'S ECTOPIC PREGNANCY.

"Some books are lies frae end to end,
And some great lies were never penn'd."

MODERN gynecology is the "Arabian Nights" of the medical literature of to-day, where Sinbad the sailor vies in rash emulation with the new Aladdin—Apostoli—and his wonderful lamp, electricity. Romance and reality run hand in hand, and what with honest error and intentional exaggeration, it is no small task to filter truth from fiction. Now and anon, there appears a book which, apart from whatever fault or deficiency it possesses, strives to separate science from imagination, and, basing conclusions upon wide experience and careful investigation, makes and marks an era in the science it elucidates. In the March number of this journal we made editorial comment upon such a book at a length justified only by the importance of the subject discussed.[1] We are led to return to the discussion by a review of the same book, that appeared in the *American Journal of the Medical Sciences* for April, 1889.

The author of this critique, "J. C. R.," evidently appreciating both the merit of the book and the position of its author, praises Cæsar before he slays him. The praise is, however, mitigated by an undertone of deprecation, which oozes clamily through the

1. Tait—"Lectures on Ectopic Pregnancy and Pelvic Hematocele."

writer's evident desire to be considered fair in his masquerade of justice. Now, as "critics all are ready made," so our reviewer has "just enough of learning to misquote, and a mind well skilled to find or forge a fault." That a proper value may be set upon the intrinsic worth of such criticism from such a source as "J. C. R.," whoever he may be, we will first devote our attention to his errors of statement concerning the book upon which, like Don Quixote upon the wind-mill, he charges.

Of the exceptions taken to the book, the following is so puerile that, save to show the animus and ability of the reviewer "to forge a fault," it should receive no attention. "J. C. R." says: "The title of the work, so far as 'lectures' are concerned, is a misnomer. No address heads the opening, no statement is made as to when, where, or to whom was the delivery. There is no division in the text; the book begins without preface or introduction and continues unbroken to the end." Now, as Mr. Tait is Professor of Gynecology in Queen's College, Birmingham, it takes only a modicum of critical acumen to divine where the "lectures" were delivered. A little further attention would have discovered that in the *British Medical Journal* of September 1, 1888, is published "A Lecture on a Case of Extra-Uterine Pregnancy," in which Mr. Tait's scheme of ectopic gestation is given exactly as in the volume now under consideration. Mr. Tait's data are so peculiarly his own, that it would seem rather strange were he not to utilize them as lectures in the position he holds. Such carping should have no place in intelligent criticism. Further, page 379, we find the following: "We have only here to comment upon one point which is . . . . . . with due regard to the honor of this country, we can let no account pass unchallenged that does not contain some mention of the work of Stephen Rogers." On page 23 of Mr. Tait's book is the following, quoted from Parry: "The first suggestion of performing gastrotomy to save a woman dying of early rupture of the cyst, came, so far as we know, from our countryman Dr. Harbert, while to Rogers belongs the credit of formulating the arguments in favor of this practice and bringing them prominently before the profession."

"J. C. R.'s" eagle-scream is, after all, the goose's hiss, at which the ghost of Stephen Rogers must smile. History repeats itself. Rome must be saved. Pluming his pinions, and screaming his disgust, he wails : . . . . "it looks very much as if fealty to Mr. Tait, rather than scientific value, was the criterion of quotation from American writers." Such exceeding grief should not be wasted on a cenotaph. Perhaps the accomplished president of the Philadelphia Obstetrical Society could explain to "J. C. R." why the American writer in question was quoted at such length by Mr. Tait.

Proceeding now to errors of a different character, we find on the same page, 379, the following : "On the treatment of the sac before rupture, of course there is nothing to be found here, as the author has never made a diagnosis. . . . He does not mention Veit's two cases, in one of which a diagnosis was made before operation, nor Price's case in this country, also with a diagnosis. Nor does he enter anywhere upon the technique of the operation ; an omission which is to be regretted." If in two cases of ectopic gestation one only is diagnosticated, the logic that pronounces such diagnosis guess-work will be accepted by most minds, and create a suspicion that a guess in one case was happily right, and in the other happily not ventured. To this we shall recur later. As to "Price's Case," (Annals of Gynecology, March, 1888,) "J. C. R." should be more definite. Of four cases there reported, the diagnosis was made in none of three previous to rupture. In a fourth case, diagnosis previous to operation was not made at all. Before witnesses are summoned, it is well to know to what they will testify. Dr. Price holds unequivocally that the diagnosis of extra-uterine pregnancy, previous to rupture, is merely a guess, and after this stage there is no dispute as to diagnosis.

On page 380, quoting three lines in reference to the operative procedure, "J. C. R." says these lines alone contain all that is said of the method of operating. On page 95 of

Mr. Tait's lectures is a discussion of the technique, extending into page 96, covering in all nearly a page and a half. Did our critic read the book he misrepresents?

The strictly scientific portions of this unique review will be considered separately in our next issue.

